# Supplementary material for: Complexome profiling of the Chlamydomonas psb28 mutant reveals TEF5 as an early PSII assembly factor
Source: Plant Cell. 2025 Mar 17;37(6):koaf055. doi: 10.1093/plcell/koaf055 (PMC12164590; doi:10.1093/plcell/koaf055)
Supplement: koaf055_Supplementary_Data [file koaf055_supplementary_data.zip › psb28_interactive.zip!/plots/intensity/qualityPlots/peptides/LHCBM3_Cre04.g232104.t1.1.html]

### Cre04.g232104.t1.1

Trivial name: LHCBM3  
SeqCoverage [median]: 61.50  
Mass: 27.38 kDa  
  
 

| Sequence | MissedCleavage | Mass [Da] | wtIntensity | muIntensity | Score |
| --- | --- | --- | --- | --- | --- |
| GPIQNLDDHLSNPTVNNAFAFATK | 0 | 2583.27 | 1962252.28 | 1283568.77 | 282.91 |
| QAPASSGIEFYGPNR | 0 | 1592.76 | 4659905.53 | 2649172.00 | 408.15 |
| QAPASSGIEFYGPNRAK | 1 | 1791.89 | 1508.57 | 0.00 | 52.03 |
| SGTQFGEAVWFK | 0 | 1355.65 | 1493691.67 | 451219.05 | 303.31 |
| TAAKQAPASSGIEFYGPNR | 1 | 1963.98 | 11502.42 | 3774.88 | 123.55 |
|  |  |  | 8128860.5 | 4387734.7 |  |
